# Identification of the Caprine Keratin-Associated Protein 20-2 (KAP20-2) Gene and Its Effect on Cashmere Traits

**DOI:** 10.3390/genes8110328

**Published:** 2017-11-17

**Authors:** Jiqing Wang, Longjie Che, Jon G. H. Hickford, Huitong Zhou, Zhiyun Hao, Yuzhu Luo, Jiang Hu, Xiu Liu, Shaobin Li

**Affiliations:** 1 Gansu Key Laboratory of Herbivorous Animal Biotechnology, Faculty of Animal Science and Technology, Gansu Agricultural University, Lanzhou 730070, China; wangjq@gsau.edu.cn (J.W.); chelongjie123@163.com (L.C.); Jon.hickford@lincoln.ac.nz (J.G.H.H.); zhouh@lincoln.ac.nz (H.Z.); hzy18298352964@163.com (Z.H.); huj@gsau.edu.cn (J.H.); liuxiu@gsau.edu.cn (X.L.); lisb@gsau.edu.cn (S.L.); 2 International Wool Research Institute, Gansu Agricultural University, Lanzhou 730070, China; 3 Gene-Marker Laboratory, Faculty of Agriculture and Life Sciences, Lincoln University, Lincoln 7647, New Zealand

**Keywords:** cashmere fibre weight, keratin-associated protein 20-2 (KAP20-2) gene, length of the curly fibre, Longdong cashmere goat, tissue expression, variation

## Abstract

The gene encoding the high glycine/tyrosine keratin-associated protein 20-2 (KAP20-2) gene has been described in humans, but has not been identified in any livestock species. A search for similar sequences in the caprine genome using the human KAP20-2 gene (*KRTAP20-2*) revealed a homologous sequence on chromosome 1. Three different banding patterns representing distinct sequences (*A*–*C*) in Longdong cashmere goats were identified using polymerase chain reaction-single stranded conformational polymorphism (PCR-SSCP) analysis. These sequences shared high sequence similarity with the human and mouse *KRTAP20-2* sequences, suggesting that *A*–*C* are caprine variants of the human and mouse genes. Four single nucleotide polymorphisms (SNPs) were identified, and three of them were non-synonymous. *KRTAP20-2* was found to be expressed in secondary hair follicles, but not in heart, liver, lung, kidney, spleen, or *longissimus dorsi* muscle. The presence of *A* was associated with increased cashmere fibre weight, while the presence of *B* was associated with a decrease in cashmere fibre weight and curly fibre length. Goats with genotype *AA* had a higher cashmere fibre weight and a higher curly fibre length than those with genotypes *AB* or *BB*. These results indicate that caprine *KRTAP20-2* variation may have value as a genetic marker for improving cashmere fibre weight.

## 1. Introduction

Cashmere goat fibre is a heterogeneous fleece comprised of wool and cashmere fibres, which are produced by the primary and secondary hair follicles, respectively. Due to its characteristics of being finer, lighter, softer, stronger, and having better elasticity and insulating properties, cashmere prices are typically more stable and higher than wool and mohair prices, and it is therefore considered a luxury product. Of all the cashmere traits of value, weight and mean fibre diameter are the most important traits economically, and they underpin most of the commercial return to cashmere producers [1]. It is well known that variation in cashmere traits is controlled by both genetic and environmental factors, so the identification of genes that regulate cashmere quantity and quality offers an opportunity to improve cashmere production.

Morphologically, cashmere fibre is composed of an external cuticular sheath and an inner cortex. Keratins and keratin-associated proteins (KAPs) are believed to play an important role in defining the physico-mechanical properties of the fibre, and fibre growth involves the expression of both the keratin and KAP genes [2]. 

In sheep, the KAPs can be classified into three broad groups according to their amino acid composition: the high sulphur (HS; ≤30 mol% cysteine), the ultra-high sulphur (UHS; >30 mol% cysteine) and the high glycine/tyrosine (HGT; 35–60 mol% glycine and tyrosine) KAPs [3]. To date, over 100 KAPs have been identified across species, and they have been categorized into 27 families [4]. 

Up to now, research into the KAP genes (*KRTAPs*) has been primarily undertaken in humans and sheep. For example, at least 80 *KRTAPs* have been categorised into 25 families in humans [5,6]. In sheep, 29 *KRTAPs* from 13 families have been identified [3,7,8]. Only 10 KAP genes have been described in goats, despite there being 17 caprine *KRTAP* sequences in the NCBI GenBank database. The published caprine *KRTAP* sequences include *KRTAP1-1* [9], *KRTAP1-4* [10], *KRTAP6-2* [11], *KRTAP7-1* [12], *KRTAP8-1* [13], *KRTAP8-2* [12], *KRTAP9-2* [14], *KRTAP11-1* [15], *KRTAP13-1* [16], and *KRTAP13-3* [9]. 

Although only a few *KRTAPs* have been identified in goats, two *KRTAPs* (*KRTAP13-1* and *KRTAP8-2*) have been reported to be associated with cashmere quality and quantity traits, including fibre diameter, weight, and the length of the curly fibre [16,17]. In the closely related species *Ovis aries* (sheep), more studies of *KRTAP* variation have been undertaken [3,18], and its association with wool traits has been described, including associations between variation in *KRTAP1-2* [19], *KRTAP6-1* [20,21], *KRTAP6-3* [22], *KRTAP8-2* [23], and *KRTAP22-1* [7], and variation in commercially important wool traits. This suggests that the identification of new caprine *KRTAPs*, variation in these genes, and any effect of observed variation on cashmere traits are worthy of investigation. 

The KAP20 family has been described in humans [24]. It consists of two members, KAP20-1 and KAP20-2, and the genes producing these KAPs are expressed in the matrix and pre-cortex of developing hair fibres [25]. In this study, we describe the identification of a caprine KAP20 gene, and reveal variation in that gene using polymerase chain reaction-single stranded conformational polymorphism (PCR-SSCP). We also confirm the expression of this gene using reverse transcription-PCR (RT-PCR), and investigate associations between variation in the gene and variation in cashmere traits in Longdong cashmere goats. 

## 2. Materials and Methods

### 2.1. Animals, Animal Tissues and Data Collected

All animal work was conducted according to the guidelines for the care and use of experimental animals established by the Ministry of Science and Technology of the People’s Republic of China (Approval number 2006–398), and was approved by the Animal Care Committee of Gansu Agricultural University.

A total of 373 Longdong cashmere goats from the progeny of 11 unrelated sires were investigated. The goats were reared at the Yusheng Cashmere Goat Breeding Company in Huan County, Gansu Province. At 12 months of age (first combing), the combed cashmere weight and the length of the curly cashmere fibre from the mid-side region were measured. Samples were also collected from the mid-side region to enable measurement of the mean fibre diameter of the fibre at the Inner Mongolia Agricultural University, Inner Mongolia, China. Blood samples from these goats were collected onto Munktell TFN paper (Munktell Filter AB, Falun, Sweden). 

Three separate three-year-old Longdong cashmere goats in the catagen phase of fibre growth were slaughtered, and tissue samples were collected from each, including samples of skin, heart, liver, lung, kidney, spleen, and *longissimus dorsi* muscle. The tissue samples were immediately frozen in liquid nitrogen and stored at −80 °C. Secondary hair follicles were separated from the skin tissue using the method described by Jin et al. [12].

### 2.2. Search for Caprine Sequences Homologous to the Human KAP20-2 Gene

The coding region of a human *KRTAP20-2* sequence (GenBank accession no. NM_181616) was used to BLAST search the caprine genome assembly GCF_001704415.1 [26]. The sequence that shared the greatest similarity with the human *KRTAP20-2* sequence was presumed to be caprine *KRTAP20-2*.

### 2.3. PCR-SSCP Analysis of Caprine KRTAP20-2

The goat genome sequence was used to design PCR primers (Table 1). The entire coding region of the putative caprine *KRTAP20-2* was amplified. These primers were synthesized by Takara Biotechnology Company Limited (Dalian, China). Goat genomic DNA for PCR amplification was purified from 1.2 mm punches of dried blood spots collected on TFN cards using a two-step washing procedure [27]. Amplifications were performed in a 20-μL reaction consisting of the genomic DNA purified from one 1.2-mm punch of dried blood, 2.0 μL of 10 × PCR buffer (Supplied with the DNA polymerase enzyme), 0.25 μM of each primer, 150 μM of each deoxynucleotides (dNTPs) (Takara), 2.5 mM Mg^2+^, 0.5 U of Taq DNA polymerase (Takara), and double-distilled water (ddH_2_O) to make up the volume. The thermal profile consisted of 2 min at 94 °C, followed by 35 cycles of 30 s at 94 °C, 30 s at 59 °C, and 30 s at 72 °C, with a final extension of 5 min at 72 °C. Amplification was carried out in Bio-Rad S1000 thermal cyclers (Bio-Rad, Hercules, CA, USA).

A 0.7-μL aliquot of each amplicon was mixed with 7 μL of loading dye (98% formamide, 10 mM ethylenediaminetetraacetic acid (EDTA), 0.025% bromophenol blue, 0.025% xylene-cyanol), and after denaturation at 95 °C for 5 min, samples were rapidly cooled on wet ice, and then loaded on 16 cm × 18 cm, 12% acrylamide:bisacrylamide (37.5:1) (Bio-Rad) gels. Electrophoresis was performed using Protean II xi cells (Bio-Rad) for 17 h in 0.5 × TBE at 210 V and 16.5 °C. The gels were silver-stained according to the method of Byun et al. [28].

### 2.4. Sequencing of Alleles and Sequence Analyses

Amplicons that were identified as homozygous by SSCP were directly sequenced in both directions at the Beijing Genomics Institute, Beijing, China. Alleles that were only in a heterozygous form were sequenced using an approach described by Gong et al. [29]. Briefly, a band corresponding to the allele was excised as a gel slice from the polyacrylamide gel, macerated, and then used as a template for re-amplification with the original primers. This second amplicon was then sequenced directly. 

Sequence alignments, translations, and comparisons were carried out using DNAMAN version 5.2.10 (Lynnon BioSoft, Vaudreuil, QC, Canada). The BLAST algorithm was used to search the NCBI GenBank (http://www.ncbi.nlm.nih.gov/) databases. Potential phosphorylation sites were predicted using the NetPhos 3.1 Server [30], and a phylogenetic tree was constructed using the predicted amino acid sequences of the three new goat *KRTAP20-2* sequences and other HGT–KAPs, including: NM_001193399 (sheep KAP6-1), KT725832 (sheep KAP6-2), NM_181604 (human KAP6-2), KT725837 (sheep KAP6-3), KT725840 (sheep KAP6-4), KT725845 (sheep KAP6-5), AY510121 (goat KAP7-1), X05638 (sheep KAP7-1), NM_181606 (human KAP7-1), AY510122 (goat KAP8-1), X05639 (sheep KAP8-1), NM_175857 (human KAP8-1), AY510123 (goat KAP8-2), KF220646 (sheep KAP8-2), NM_181607 (human KAP19-1), NM_181609 (human KAP19-3), NM_181610 (human KAP19-4), NM_181611 (human KAP19-5), NM_00130312 (human KAP19-6), NM_181614 (human KAP19-7), AB096964 (human KAP19-8), NM_181615 (human KAP20-1), NM_181616 (human KAP20-2), NM_001163615 (mouse KAP20-2), NM_181619 (human KAP21-1), NM_181617 (human KAP21-2), KX377616 (sheep KAP22-1), and NM_181620 (human KAP22-1), using MEGA version 7.0.

### 2.5. Expression of Caprine KRTAP20-2 in Selected Tissues

Total RNA from the seven tissue samples collected was extracted using TRIzol reagent (Invitrogen, Carlsbad, CA, USA), and the quality and concentration of RNA extracted were checked using 2% agarose gels electrophoresis and UV spectrophotometry. Reverse transcription was performed to produce cDNA using the PrimeScript™ RT Reagent Kit with gDNA Eraser (Perfect Real Time) (Takara), and following the manufacturer’s instructions. The amplification of the cDNA was carried out using another set of PCR primers located within the *KRTAP20-2* coding region (TGGAAACTACTATGGCGGCC and TATCTTCTGCAACAGGATGG; Table 1). This enabled amplification of a shorter fragment. This amplification used the same conditions and thermal profile described above for the genomic amplification, but the genomic DNA was replaced by 0.8 μL of the cDNA. The goat β-actin gene was chosen as an internal reference standard, with the PCR primers for the amplification of this sequence described in Table 1. PCR products were examined by electrophoresis in 1.0% agarose gels. 

### 2.6. Statistical Analyses

All analyses were performed using IBM SPSS Statistics version 24.0 (IBM, New York, NY, USA). General linear mixed-effects models (GLMMs) were used to assess whether the presence or absence (coded as 1 or 0 respectively) of *KRTAP20-2* alleles was associated with various cashmere traits in the 373 Longdong cashmere goats studied. For genotypes with a frequency >5% (thus providing adequate sample size), a second set of GLMMs were used to ascertain the effect of genotype on various cashmere traits. To reduce the probability of false positive results during the multiple comparisons in these models, a Bonferroni correction was applied. Sire and gender were found to affect (*p* < 0.05) of all the fibre traits, so they were included in the models as a random and fixed factor, respectively. Birth rank was not found to affect cashmere fibre traits, and was not included in the models. Only the main effects were tested.

## 3. Results

### 3.1. Identification of Caprine KRTAP20-2

**Figure 1:**
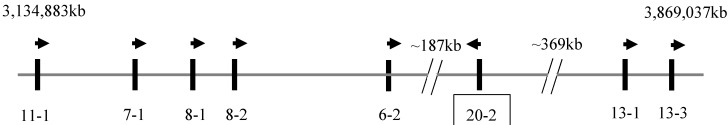
Location of the putative *KRTAP20-2* (boxed), together with seven other *KRTAPs* on goat chromosome 1. The vertical bars represent the keratin-associated protein (KAP) genes, and the arrows indicate the direction of transcription. The numbers below these bars are the KAP gene names (e.g., 11-1 represents *KRTAP11-1*). The spacing of the genes is only approximate, and is based on the caprine genome assembly, as are the nucleotide coordinates [26].

A BLAST search of the caprine genome assembly GCF_001704415.1 using the human *KRTAP20-2* coding sequence (NM_181616) revealed a region on goat chromosome 1 (nt 3486283_3486471) that contained a 189-bp open reading frame, and that had 75% nucleotide identity with the human *KRTAP20-2* sequence. Seven previously described caprine KAP genes were also identified near this region; these were *KRTAP11-1*, *KRTAP7-1*, *KRTAP8-1*, *KRTAP8-2*, *KRTAP6-2*, *KRTAP20-2*, *KRTAP13-1*, and *KRTAP13-3*, in order from the centromere to the telomere (Figure 1).

### 3.2. Detection of Allelic Variation in Caprine KRTAP20-2

**Figure 2:**
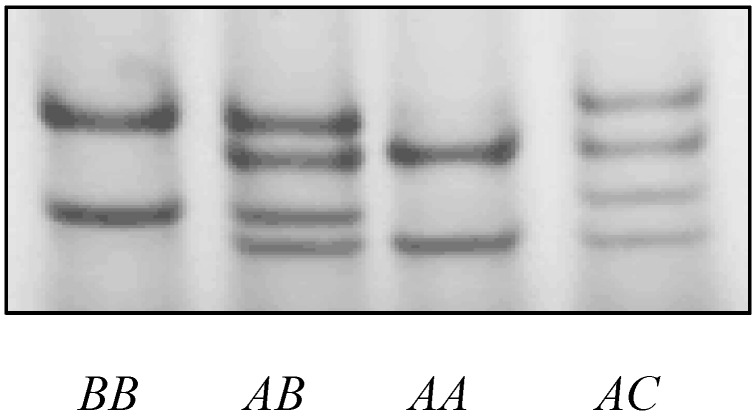
Representative PCR-SSCP patterns for the caprine KAP20-2 gene. Four genotypes are shown, and there are three unique banding patterns, which correspond to three alleles *A–C*.

Amplicons of the predicted size (273 bp) were obtained using the SSCP analysis of DNA in the goat blood samples. Three different PCR-SSCP patterns were detected (Figure 2). Either one pattern, or a combination of two patterns, was observed for each goat. Sequencing of amplicons representing the three unique SSCP patterns, revealed three different alleles (named *A* to *C*), which differed at the nucleotide level within the 189-bp coding sequence. Allele *B* was identical to the caprine genome assembly, while the *A* and *C* alleles differed from the deposited genome sequence.

**Figure 3:**
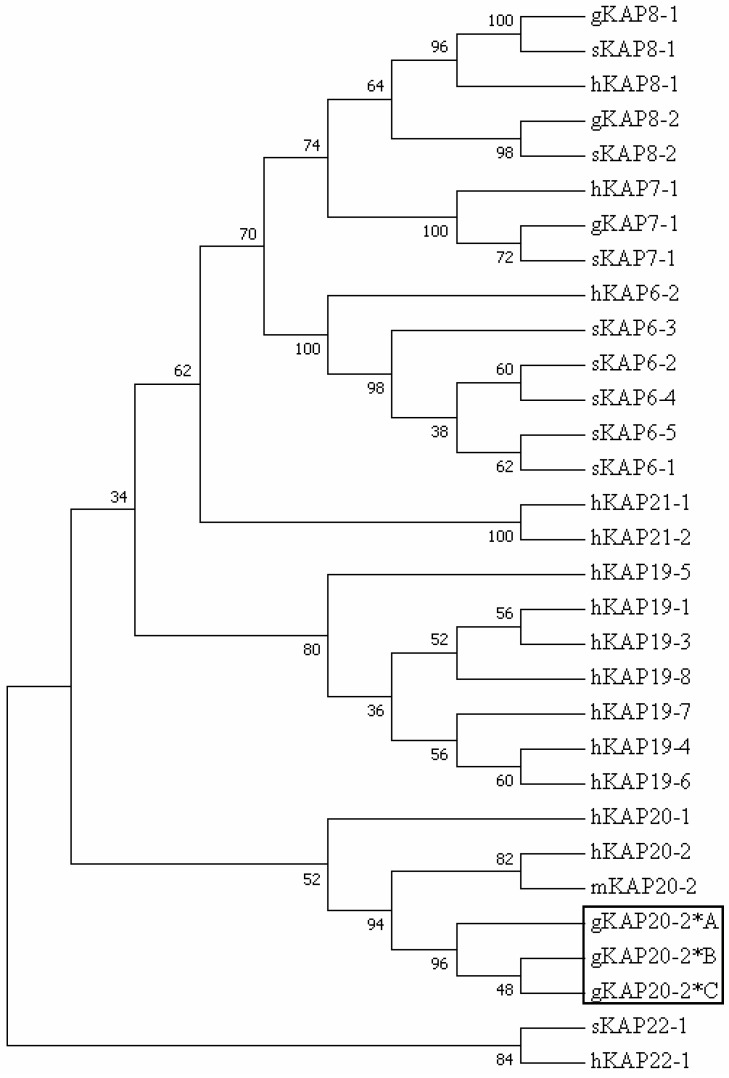
Maximum parsimony tree of the high glycine/tyrosine keratin-associated proteins (HGT-KAPs) identified in goats, sheep and humans, together with the mouse KAP20-2 sequence. The tree was constructed using the predicted amino acid sequences. The numbers at the forks indicate the bootstrap confidence values. The caprine KAPs are indicated with a prefix “g”, while the sequences of sheep, human, and mouse are indicated with “s”, “h” and “m”, respectively. The three newly identified goat KAP20-2 sequences are shown in a box, and the GenBank accession numbers for the other HGT-KAPs are: NM_001193399 (sKAP6-1), KT725832 (sKAP6-2), NM_181604 (hKAP6-2), KT725837 (sKAP6-3), KT725840 (sKAP6-4), KT725845 (sKAP6-5), AY510121 (gKAP7-1), X05638 (sKAP7-1), NM_181606 (hKAP7-1), AY510122 (gKAP8-1), X05639 (sKAP8-1), NM_175857 (hKAP8-1), AY510123 (gKAP8-2), KF220646 (sKAP8-2), NM_181607 (hKAP19-1), NM_181609 (hKAP19-3), NM_181610 (hKAP19-4), NM_181611 (hKAP19-5), NM_00130312 (hKAP19-6), NM_181614 (hKAP19-7), AB096964 (hKAP19-8), NM_181615 (hKAP20-1), NM_181616 (hKAP20-2), NM_001163615 (mKAP20-2), NM_181619 (hKAP21-1), NM_181617 (hKAP21-2), KX377616 (sKAP22-1), and NM_181620 (hKAP22-1).

Phylogenetic analysis revealed that the predicted amino acid sequences of the DNA sequences identified were more closely related to KAP20-2 sequences from human and mouse than other HGT-KAP sequences that have been identified in goats, sheep, and humans (Figure 3). This suggests that these goat sequences represent alleles of caprine *KRTAP20-2*. The alleles were named CAPHI-KRTAP20-2*A to CAPHI-KRTAP20-2*C according to the nomenclature proposed by Gong et al. [31], and the sequences were deposited in GenBank with accession numbers MF973462–MF973464, respectively. 

Four single nucleotide polymorphisms (SNPs) (c.27C>T, c.37C>T, c.125T>C, and c.126G>A) were identified among the three sequences. These SNPs were all located in the coding sequence, and three of them were non-synonymous. SNP c.37C>T would result in a putative amino acid change of p.His13Tyr, whereas the other two non-synonymous SNPs (c.125T>C and c.126G>A) were located within the same codon, and would result in an amino acid change of p.Met42Thr (Table 2).

### 3.3. Amino Acid Sequence Analyses

**Figure 4:**
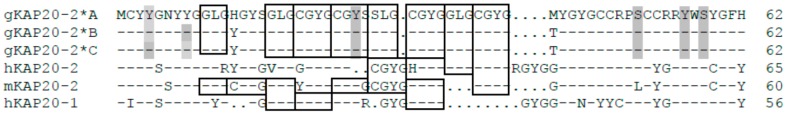
Alignment of KAP20-n sequences from goat, human, and mouse. The amino acid sequences are predicted from the nucleotide sequences, and are shown in one-letter code. Dashes represent amino acids identical to the top sequence, and dots have been introduced to improve the alignment. The length of each sequence is shown on the right. Residues that may be phosphorylated in the caprine KAP20-2 sequences are shaded. Boxes indicate the repeats of (G/S)LG and CGY(G/S). The goat sequences are indicated with a prefix “g”, while the sequences of human and mouse are indicated with “h” and “m”, respectively.

The three caprine *KRTAP20-2* sequences would all encode polypeptides of 62 amino acid residues. These polypeptides contained a high content of glycine (32.26%), and moderate levels of tyrosine (20.97–22.58%) and cysteine (14.52%). The theoretical isoelectric points (pI) of the three putative polypeptides were all 7.26 and for these notional caprine KAP20-2 polypeptides, between five and six residues were predicted to be potentially phosphorylated (Figure 4).

### 3.4. Expression of KRTAP20-2 in Different Tissues

**Figure 5:**
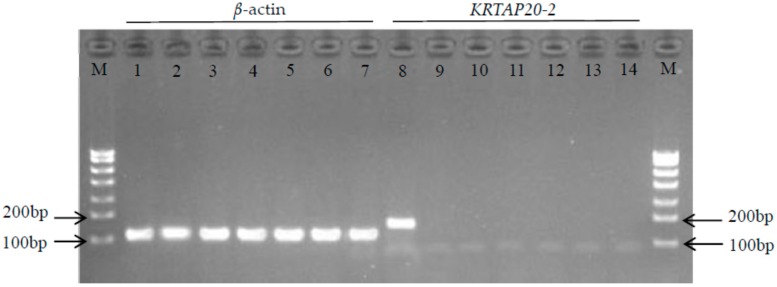
RT-PCR results of β-actin and *KRTAP20-2* in seven tissues of Longdong cashmere goats. M: marker. Lanes 1–7 and 8–14 are expression results of β-actin and *KRTAP20-2* in seven tissues, respectively. Numbers below lanes are tissues studied: 1 and 8: secondary hair follicle; 2 and 9: heart; 3 and 10: liver; 4 and 11: lung; 5 and 12: kidney; 6 and 13: spleen; 7 and 14: *longissimus dorsi* muscle.

The RT-PCR analysis of *KRTAP20-2* expression in the seven different tissues retrieved from the Longdong cashmere goats revealed that the gene was expressed at high levels in secondary hair follicles, but expression was not detected in the other six tissues (heart, liver, lung, kidney, spleen, and *longissimus dorsi* muscle) (Figure 5).

### 3.5. Phenotypic Correlations between the Various Cashmere Traits

Cashmere weight had a moderately high positive correlation (0.3 < |*r*| ≤ 0.7) with the length of the curly fibre (*r* = 0.490), while mean fibre diameter had a weak positive correlation (|*r*| ≤ 0.3) with cashmere weight (*r* = 0.280) and the length of the curly fibre (*r* = 0.216) (Table 3).

### 3.6. Allele and Genotype Frequencies of KRTAP20-2 in the Longdong Cashmere Goats

The frequencies of the three *KRTAP20-2* alleles in the 373 Longdong cashmere goats were: *A*: 68.37%, *B*: 28.95%, and *C*: 2.68%. Five genotypes (*AA*, *AB*, *BB*, *AC*, and *BC*) were detected. Of these, *AA*, *AB*, and *BB* were the most common, with a combined frequency of 94%. The remaining two genotypes (*AC* and *BC*) occurred at a frequency less than 5%, and genotype *CC* was not observed. 

### 3.7. Associations between KRTAP20-2 Variation and Cashmere Traits

Of the three alleles detected in the Longdong cashmere goats, allele *C* was present at a frequency of less than 5%; given this low frequency and potential for bias, its association with cashmere traits was not investigated. In the presences/absence models, the presence of *A* was associated with increased cashmere fibre weight (present: 416 ± 2.8 g; absent: 378 ± 5.4 g; *p* < 0.001), while the presence of *B* was found to be associated with a decrease in cashmere fibre weight (present: 392 ± 3.8 g; absent: 422 ± 3.1 g; *p* < 0.001) and the length of the curly fibre (present: 4.1 ± 0.04 cm; absent: 4.2 ± 0.04 cm; *p* = 0.021). No association with fibre diameter was detected for either *A* or *B* (Table 4).

For *AA*, *AB*, and *BB* goats, genotype was found to have an effect on some cashmere traits. Goats with genotype *AA* had a higher cashmere fibre weight (*p* < 0.001), and the length of the curly fibre (*p* = 0.032). No associations were found between the *KRTAP20-2* genotype and mean fibre diameter (Table 5). 

## 4. Discussion

This study reports the identification of a new caprine KAP gene encoding a HGT-KAP protein, and the association between variation in the sequence of that gene, and some cashmere fibre traits. The putative caprine *KRTAP20-2* was located at a previously unannotated position of chromosome 1, and it shared the highest homology with the *KRTAP20-2* sequences from human and mouse. Based on this, it was concluded that it represents the caprine *KRTAP20-2* sequences. The identification of *KRTAP20-2* brings the total number of caprine KAP genes described in the published literature from 10 to 11. A number of ovine *KRTAPs* have been identified using a similar investigative approach, including *KRTAP8-2* [32], *KRTAP15-1* [8], *KRTAP22-1* [7], and *KRTAP24-1* [33], and this suggests that the approach described in the study is robust as regards identifying *KRTAPs* in goats as well as sheep.

In the tissues investigated in the study, *KRTAP20-2* mRNA was only identified in secondary hair follicles, but not in heart, liver, lung, kidney, spleen, and muscle. This is consistent with findings reported for Liaoning cashmere goats, where *KRTAP7-1* and *KRTAP8-2* were only expressed in hair follicles and not in heart, liver, spleen, lung, and kidney tissues [12], and the observation of Rogers et al. [34] that a large number of the KAP family members were exclusively expressed in hair follicles. Given that cashmere fibre is produced by secondary hair follicles, it could be inferred that the specific expression of *KRTAP20-2* in secondary hair follicles may at least in part be responsible for fibre traits.

Despite the predicted amino acid sequences of caprine *KRTAP20-2* having high similarity to *KRTAP20-2* sequences from human and mouse, some differences in the sequences exist. Firstly, the human and mouse proteins contain conserved amino-terminal and carboxyl-terminal sequences of M(I/C)YY(R/S)(G/N)YY and RY(W/-)(S/-)(Y/C)GFY) [24], whereas the goat KAP20-2 sequences described here do not precisely contain these conserved sequences. Instead, they have the sequences MCYYGNYY and RYWSYGFH at the amino-terminal and carboxyl-terminal ends, respectively (Figure 4). There is also variability in the number of sequence repeats in the putative amino acid sequences. In the middle region of the human and mouse protein sequence, there is a trimer repeat (G/S)LG and a tetramer repeat CGY(G/S). The goat sequence contains four repeats of (G/S)LG, whereas the human and mouse KAP20-2 sequences have one and two repeats, respectively (Figure 4). The goat sequences also contain four repeats of CGY(G/S), but human KAP20-2 has two repeats, and mouse KAP20-2 has five repeats (Figure 4). 

The putative goat KAP20-2 sequences have a lower content of glycine and tyrosine (53.23–54.84 mol%) than human KAP20-2 (61.5 mol%) and mouse KAP20-2 (63.3 mol%). However, the serine content in goat (8.1 mol%) is higher than that in the human (4.6 mol%) and mouse (3.3 mol%) proteins. The cysteine content in goat (14.5 mol%) is also higher than in human (12.3 mol%), but lower than in mouse (16.7 mol%). Finally, human KAP20-2 is the longest polypeptide with 65 amino acids, followed by the goat sequence, with 62 amino acids, and 60 amino acids for the mouse. 

It is interesting to note that some of the putative amino acid changes in KAP20-2 would result in a change in the number of potential phosphorylation sites. Although the presence and function of phosphorylation is poorly understood for KAPs, it is known to occur for the keratins, and affects keratin assembly and organization [35]. This in turn influences the resulting fibre structure. If variation in phosphorylation patterns occur, then it is possible that this may influence cashmere traits. 

It is noteworthy that four SNPs were detected in the coding sequence of caprine *KRTAP20-2*, and that most (three out of four) of them were non-synonymous substitutions. When the DNA sequences were compared, it was found that all of the three non-synonymous SNPs (c.37C>T, c.125T>C, and c.126G>A) were in complete linkage, and what is more, two of these SNPs were adjacent and located within the same codon (Table 2). This suggests that these SNPs may have co-evolved, and then been maintained in goats. The co-evolution of SNPs has been observed in the *KAP15-1* gene in sheep, and has been suggested to result from gene conversion or non-reciprocal genetic exchange [8]. Further research is needed on the evolution of the KAP genes in ruminants. 

The content of HGT-KAPs in fibre vary both between and within species, ranging from more than 30% in echidna quill and 18% in mouse hair to less than 12% in sheep wool. There is less than 1% HGT-KAP in Lincoln sheep wool, and between 4–12% in Merino wool. The wide range in the content of the HGT-KAPs in different type of fibres and breeds of sheep suggests that these proteins may be responsible for some of the variation in fibre properties, which was in part confirmed in this study. Of the three cashmere traits studied, variation in caprine *KRTAP20-2* was associated with the combed fibre weight and the length of the curly fibre, but not the mean fibre diameter. The differing effect of the gene on the traits may be a consequence of phenotypic correlations between the traits. These phenotypic correlations are similar to the findings reported by Bishop et al. [36], Zhou et al. [37], and Ma et al. [38], who found moderate correlations between combed cashmere weight and the length of the fibre, mean fibre diameter and combed cashmere weight, and mean fibre diameter and the length of the fibre. 

The effect of *KRTAP20-2* on cashmere fibre traits is similar to that reported for *KRTAP8-2* [17] and *KRTAP13-1* [16] in Inner Mongolian cashmere goats and Xinjiang cashmere goats, respectively. Given that these gene are clustered on goat chromosome 1, and that *KRTAP20-2* and *KRTAP8-2* are HGT-KAP genes, whereas *KRTAP13-1* is a HS-KAP gene, the possibility exists that the functional effect detected for these genes may due to tight linkage to other KAP genes. This would require further investigation of other linked KAP genes on the same chromosome. 

The effect of the presence of *A* on cashmere fibre weight in these goats was large, and it suggests that selection for *A* would be economically valuable. Goats with the genotype *AA* could notionally increase cashmere fibre weight by 12.5% compared with those with genotype *BB* (estimated from Table 5). Thus, for a goat with 400 g of combed cashmere fibre weight, the weight improvement might equate to approximately an extra 50 g of cashmere fibre, and without any significant change in fibre diameter. Therefore, *KRTAP20-2* may be a useful genetic marker for improved cashmere goat breeding. Further investigation on more goats from different breeds is needed to confirm this finding, as it has been reported that the phenotypic correlations between cashmere fibre traits vary between goats of differing origin [39]. 

## Figures and Tables

**Table 1:** PCR primers used in this study.

Gene	Sequence (5′-3′)	Amplicon Size (bp)	Purpose of Primers
*KRTAP20-2*	CAGACTATAGAGACAGATTCC CCAATTAGTTGAGTTTCTCTG	273	Gene identification and SSCP analysis
*KRTAP20-2*	TGGAAACTACTATGGCGGCC TATCTTCTGCAACAGGATGG	156	Reverse transcription (RT)-PCR
β-actin	AGCCTTCCTTCCTGGGCATGGA GGACAGCACCGTGTTGGCGTAGA	113	RT-PCR

bp: base pairs; SSCP: single stranded conformational polymorphism.

**Table 2:** Nucleotide substitution and alleles of the caprine KAP20-2 gene.

Substitution ^1^	Allele			Amino Acid Change ^1^
	*A*	*B*	*C*	
c.27C>T	C	T	C	Silent
c.37C>T	C	T	T	p.His13Tyr
c.125T>C	T	C	C	p.Met42Thr
c.126G>A	G	A	A	p.Met42Thr

^1^ Numbering of nucleotide and amino acid positions follows the guidelines of the Human Genome Variation Society (HGVS) nomenclature (http://varnomen.hgvs.org/).

**Table 3:** Pearson correlation coefficients, *r*, were calculated to test the strength of the associations between the various traits.

**Trait**	Cashmere fibre weight	Mean fibre diameter
Mean fibre diameter	0.280, *p* < 0.001	
Curly fibre length	0.490, *p* < 0.001	0.216, *p* < 0.001

**Table 4:** Association of *KRTAP20-2* alleles with various cashmere traits (Mean ± SE) ^1^ in Longdong cashmere goats.

Cashmere Trait (Unit)	Allele	Absent		Present		*p* Value ^1^
		Mean ± SE	*n*	Mean ± SE	*n*	
Cashmere fibre weight (g)	*A*	**378 ± 5.4**	64	**416 ± 2.8**	309	**<0.001**
	*B*	**422 ± 3.1**	218	**392 ± 3.8**	155	**<0.001**
Mean fibre diameter (μm)	*A*	13.6 ± 0.06	64	13.6 ± 0.03	309	0.581
	*B*	13.6 ± 0.03	218	13.6 ± 0.04	155	0.748
Curly fibre length (cm)	*A*	4.1 ± 0.06	64	4.2 ± 0.03	309	0.328
	*B*	**4.2 ± 0.04**	218	**4.1 ± 0.04**	155	**0.021**

^1^ Estimated marginal means and standard errors (SE) of those means derived from general linear mixed-effects models that included “gender” as a fixed factor, and “sire” as a random factor. *p* < 0.05 are in bold.

**Table 5:** The effect of the *KRTAP20-2* genotype on various cashmere traits (Mean ± SE) ^1^ in Longdong cashmere goats.

Cashmere Trait (Unit)	Mean ± SE			*p* Value
	*AA* (*n* = 201)	*AB* (*n* = 91)	*BB* (*n* = 61)	
Cashmere fibre weight (g)	**422 ± 3.2 ^a^**	**402 ± 4.6 ^b^**	**375 ± 5.5 ^c^**	**<0.001**
Mean fibre diameter (μm)	13.6 ± 0.03	13.6 ± 0.05	13.6 ± 0.06	0.793
Curly fibre length (cm)	**4.2 ± 0.04 ^a^**	**4.1 ± 0.05 ^b^**	**4.1 ± 0.06 ^b^**	**0.032**

^1^ Estimated marginal means and SE of those means derived from general linear mixed-effects models that included “gender” as a fixed factor and “sire” as a random factor. A Bonferroni correction was applied to correct for multiple comparisons. Means within rows that do not share a superscript letter (a, b or c) are significantly (*p* < 0.05) different and bolded.
